# Biodegradable and Peroxidase‐Mimetic Boron Oxynitride Nanozyme for Breast Cancer Therapy

**DOI:** 10.1002/advs.202101184

**Published:** 2021-06-30

**Authors:** Lula Zeng, Yuxin Han, Zhiwei Chen, Kang Jiang, Dmitri Golberg, Qunhong Weng

**Affiliations:** ^1^ College of Materials Science and Engineering Hunan University Changsha 410082 P. R. China; ^2^ Centre for Materials Science and School of Chemistry and Physics Queensland University of Technology (QUT) Brisbane 4000 Australia

**Keywords:** biodegradable, boron nitride, breast cancer, nanozyme, peroxidase

## Abstract

Nanomaterials having enzyme‐like activities are recognized as potentially important self‐therapeutic nanomedicines. Herein, a peroxidase‐like artificial enzyme is developed based on novel biodegradable boron oxynitride (BON) nanostructures for highly efficient and multi‐mode breast cancer therapies. The BON nanozyme catalytically generates cytotoxic hydroxyl radicals, which induce apoptosis of 4T1 cancer cells and significantly reduce the cell viability by 82% in 48 h. In vivo experiment reveals a high potency of the BON nanozyme for breast tumor growth inhibitions by 97% after 14‐day treatment compared with the control, which are 10 times or 1.3 times more effective than the inert or B‐releasing boron nitride (BN) nanospheres, respectively. This work highlights the BON nanozyme and its functional integrations within the BN nanomedicine platform for high‐potency breast cancer therapies.

## Graphical abstract

An artificial peroxidase‐like nanozyme is designed based on a biodegradable boron oxynitride nanostructure, which generates hydroxyl radical efficiently and durably, reduces breast cancer cell viability by 82% in 48 h, and inhibits the cancer growth by 97% in 14 days.

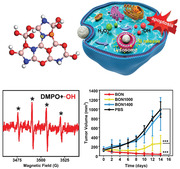

## 1 Introduction

Breast cancer is ranked as the most commonly diagnosed cancer worldwide based on the recent statistics of the World Health Organization, affecting health and lives of millions of women.^[^ ^1^ ^]^ In recent decades, great efforts have been made in the breast cancer diagnosis and therapy,^[^ ^2^ ^]^ and many strategies have been developed to prevent and treat this disease, including surgery, chemotherapy,^[^ ^3^ ^]^ radiotherapy,^[^ ^4^, ^5^ ^]^ hormone therapy,^[^ ^6^, ^7^ ^]^ and targeted therapy.^[^ ^8^, ^9^ ^]^ However, breast cancer has the characteristics of rapid growth, easy metastasis and recurrence, and generation of drug resistance after therapies,^[^ ^10^, ^11^ ^]^ making the eradication of the tumor cells and complete rehabilitation very challenging, particularly for the late‐stage patients. Besides the standard therapy strategies mentioned above, the emerging nanoscience and nanotechnology are breeding innovative diagnosis and therapy methods for tackling this cancer.^[^ ^12^, ^13^, ^14^ ^]^ To this end, many nanomaterials have been designed and implemented based on the principles of photothermal therapy,^[^ ^15^, ^16^ ^]^ photodynamic therapy,^[^ ^17^, ^18^ ^]^ magnetotherapy,^[^ ^19^, ^20^ ^]^ etc. However, these therapies rely on external stimuli, like light or field, which significantly limits the clinical translations and applications of these nanomaterials. Therefore, much attention starts to be paid on developing non‐invasive and self‐therapeutic nanomaterials that work without involvements of drugs and external stimuli such like nanozymes, that is, enzyme‐mimetic inorganic or organic nanomaterials.^[^ ^21^, ^22^, ^23^ ^]^

These artificial enzymes, particularly the peroxidase‐like nanozymes, have aroused great interest and become a new anti‐cancer methodology. Reactive oxygen species (ROS) are endogenic byproducts of cell metabolism, including singlet oxygen, superoxide anion free radicals, and hydroxyl free radicals (·OH), which play a crucial role in the process of intracellular signal transduction.^[^ ^24^ ^]^ Among the ROS species, the hydroxyl radical has a high reactivity and can cause significant damage to protein, lipid, and DNA of cells.^[^ ^25^ ^]^ The rapid proliferation of tumor cells usually leads to a large amount of hydrogen peroxide accumulated in the cells.^[^ ^26^ ^]^ Peroxidase‐mimetic nanozymes take advantage of this feature and convert hydrogen peroxide into harmful ROS, which could be utilized for novel cancer treatments. Peroxidase‐like nanomaterials, such as, ferromagnetic nanoparticles,^[^ ^27^ ^]^ porous carbon nanospheres,^[^ ^28^ ^]^ and gold nanoparticle‐loaded mesoporous silica‐coated graphene (GSF@AuNPs), can catalyze H_2_O_2_ to generate ROS like horseradish peroxidase.^[^ ^29^ ^]^ It was found that the nitrogen‐doped porous carbon nanospheres had shown multiple enzyme‐like activities and were utilized for in vivo ROS species regulations. The higher N content in the nanospheres, the higher enzyme‐like effect on inhibiting tumors was observed.^[^ ^28^ ^]^ The peroxidase‐like activity of nanoparticles may cause enhanced cleavage of nucleic acids, proteins, and polysaccharides.^[^ ^27^ ^]^ Compared with natural enzymes that would be easily denatured and degraded when enter circulation system, the artificial nanozymes have numerous advantages in higher chemical stability, much easier synthesis, and adjustable catalytic performance, making them competent for in vivo catalysis.^[^ ^30^ ^]^

Hexagonal boron nitride (*h*‐BN), a structural analog of graphite, has excellent oxidation resistance, thermal and chemical stability.^[^ ^31^ ^]^ As a non‐toxic skin lightener, it has been adopted in many cosmetic products,^[^ ^32^ ^]^ and also shows tremendous potentials for biomedical applications.^[^ ^33^ ^]^ Purified *h*‐BN and BN nanomaterials have excellent biocompatibility.^[^ ^34^ ^]^ After functionalization, the hydrophobic BN structures could be changed into highly water‐soluble materials, which were employed for anti‐cancer drug delivery to achieve impressive cancer cell inhibition efficiency.^[^ ^35^ ^]^ Besides, the BN materials themselves were also explored for direct cancer therapies. For example, BN nanotubes (BNNTs) and BN nanoparticles were proven as boron neutron therapeutic agents that can effectively treat breast cancer.^[^ ^36^ ^]^ Li et al. found that hollow BN nanospheres could enduringly release boric acid for prostate cancer treatment and achieved high cancer inhibitions.^[^ ^37^ ^]^ Because these developed cancer treatments based on BN agents must rely on external stimuli or the release of therapeutic agents, it is difficult to realize the approaches in cancer therapeutic practices. However, these progresses highlight the great potential of BN materials, as an integrable nanomedicine platform, toward future multifunctional cancer diagnosis and therapy.

In this work, we have developed a brand‐new peroxidase‐mimetic nanozyme based on biodegradable boron oxynitride (BON) structure, which can in vitro and in vivo catalyze hydrogen peroxide to efficiently generate hydroxyl radicals. The BON nanospheres were synthesized via a high‐temperature pyrolysis of organic borates in ammonia. Their structures and properties were systematically analyzed employing various microscopic and spectroscopic methods. The catalytic performance of the nanozyme was proved directly by electron spin resonance (ESR) and further evaluated by a fluorescent trapping agent. The mechanism of breast cancer cell death induced by BON was evaluated by a flow cytometry. These results showed that the BON nanozyme could be degraded in 7 days in aqueous solution; they enter 4T1 cancer cell lysosomes through phagocytosis, which reduced the viability of the cancer cells by 82% in 48 h by triggering the cell apoptosis process. In vivo experiment confirmed the high potency of the BON nanozyme for breast tumor inhibitions by 97% after 14 days’ treatment compared with the control, a much higher number compared with those of the inert and B‐releasing BN nanospheres. This contribution provides a new platform for powerful nanozyme designs toward broad biomedical applications.

## 2 Results and Discussions

### 2.1 Microscopic and Spectroscopic Characterizations of Boron Oxynitride

**Figure 1:**
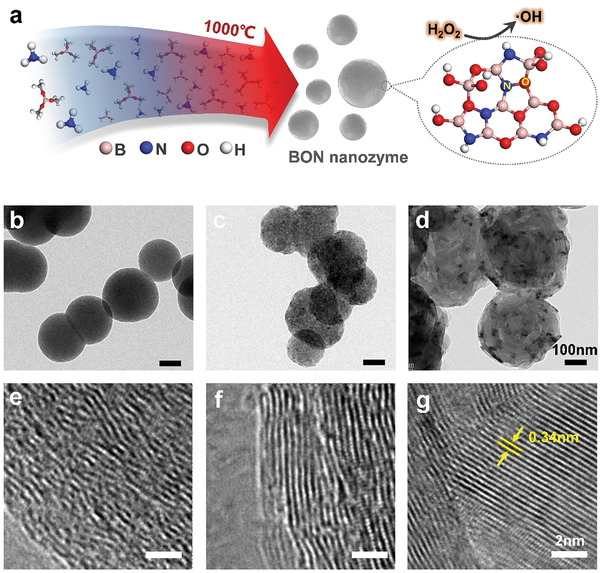
Morphological and structural analysis of BON nanospheres. a) Schematic illustration of the pyrolytic synthesis of BON nanospheres. b–d) TEM images of BON, BON1000, and BON1400. The scale bars are 100 nm. e–g) High‐magnification TEM images of BON, BON1000, and BON1400. The scale bars are 2 nm.

As shown by the scanning electron microscopy (SEM) images (Figure S2, Supporting Information) and the transmission electron microscopy (TEM) images (**Figure** **1**a,b), the pyrolytically synthesized BON has a spherical morphology with the particle size ranging from 100 to 500 nm, in agreement with the dynamic light scattering (DLS) measurement (Figure S3, Supporting Information). It exhibits excellent dispersibility in water even at a high concentration of 5 mg mL^−1^. High‐resolution TEM images reveal the amorphous structure of the BON, whose crystallinity increases along with the raise of annealing temperature, as shown in Figure 1b–g. Zeta potential measurement reveals a high and negative surface potential of −47.4 mV for the dispersed BON nanospheres (Figure S3, Supporting Information), indicating an excellent stability of the formed BON colloid solution in water. Further annealing treatment of the BON samples results in structural evolutions of the nanospheres. As indicated in Figure 1c,d, the BON1000 annealed at 1000 °C for 4 h generally maintains the sphere morphology with the formation of porous structures, while the BON1400 sample reorganizes into a crystalline and hollow structure. In this study, these BN nanomaterials are employed as the B‐releasing and inert BN nanospheres, respectively.^[^ ^37^ ^]^ Accordingly, the particle size of the BON samples determined by DLS increases after the annealing treatments along with the decrease of Zeta potential (Figure S3, Supporting Information).

**Figure 2:**
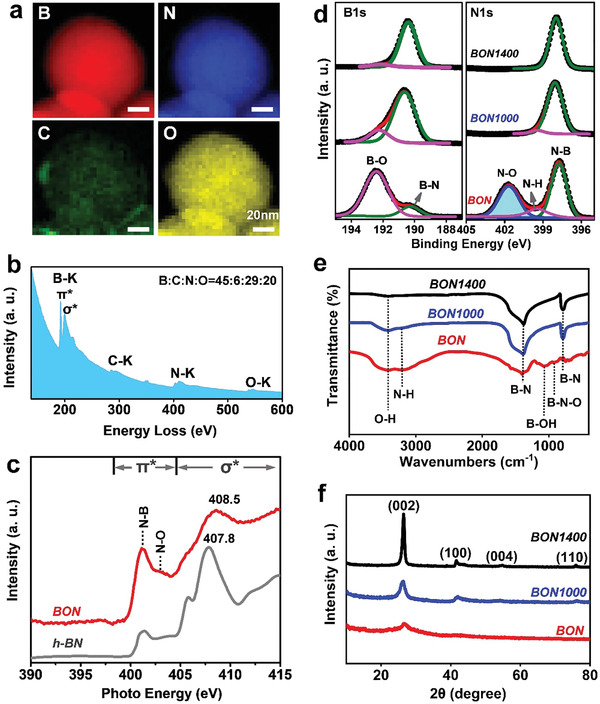
Microscopic and spectroscopic characterizations of BON, BON1000, and BON1400. a) EELS maps of B‐K, N‐K, C‐K, and O‐K of BON sample. b) EEL spectrum of BON. c) N K‐edge XANES spectrum of BON. The *h*‐BN data were reproduced from ref. ^[^ ^39^ ^]^ for comparison. d) XPS B1s and N1s spectra of BON, BON1000, and BON1400 samples. e) FTIR spectra of BON, BON1000, and BON1400. f) XRD patterns of BON, BON1000, and BON1400.

Electron energy loss spectroscopy (EELS), X‐ray absorption near edge structure spectroscopy (XANES), X‐ray photoelectron spectroscopy (XPS) and Fourier transform infrared (FTIR) spectroscopy were employed for chemical composition and chemical state analysis of the BON samples. EELS maps shown in **Figure** **2**a reveal uniform distributions of the B, N, and O elements in BON nanospheres. The corresponding EEL spectrum confirms that the main compositions are B, N, and O with a minor C component. The molar ratio of B:C:N:O in the BON is 45:6:29:20 (Figure 2b). In N K‐edge XANES (Figure 2c), the sharp peaks seen at ≈401 eV in BON and *h*‐BN are caused by the X‐ray photoexcitation of N1s electrons to π* (N‐B), while the photoexcitation band at a higher energy position of 403.0 eV for the BON sample is originated from the N1s → π* (N‐O).^[^ ^38^, ^39^ ^]^ Compared with *h*‐BN, the N1s → σ* transition in BON also shifts to higher photo energy.

Importantly, both the B1s and N1s XPS spectra reveal the distinct binding energy features of the BON sample compared with BON1000 and BON1400 (Figure 2d). In either B1s or N1s spectra, both the BON1000 and BON1400 show the main B‐N components with a minor B‐O peak at 192.2 eV in the B1s spectra or a minor N‐H peak at 399.5 eV in the N1s spectra. However, there is a prominent N‐O peak at the binding energy of 401.7 eV that appears in the BON N1s spectrum, which is seldom observed and reported for BN materials.^[^ ^40^ ^]^ From FT‐IR spectra (Figure 2e), it is concluded that all samples exhibit two prominent peaks at 780 and 1380 cm^–1^ assigned to the out‐of‐plane B‐N‐B bending vibration and in‐plane B‐N stretching vibration mode, respectively. The BON possesses broad peaks at 3200 and 3400 cm^–1^, corresponding to the N‐H and O‐H vibrations, in accordance with the XPS results. The band at 920 cm^–1^ belongs to B‐N‐O vibration,^[^ ^41^ ^]^ which diminishes in BON1000 and BON1400 samples after further annealing treatments. As shown in Figure 2f, the X‐ray diffraction (XRD) pattern of BON has two broad and weak diffraction peaks at ≈26° and ≈42° belonging to the BN(002) and BN(100) planes,^[^ ^42^ ^]^ these become shaper with the raise of annealing temperature. This indicates the formation of the *h*‐BN phase with higher crystallinity after the annealing treatments, in accordance with the TEM, XPS, and FTIR results.

The synthesized BON nanospheres without further high‐temperature annealing treatments usually have very low crystallinity. It is noted that *h*‐BN has an intrinsic planar layered structure like graphite. Deviations from this structure would result in metastable phases. The existence of high oxygen content in the BN nanospheres synthesized by the CVD reaction between B(OMe)_3_ and NH_3_ was reported.^[^ ^43^, ^44^ ^]^ Oxygen was considered as a crucial factor to form the nanosphere morphology. Further annealing treatment of the BN nanospheres in Ar atmosphere would evaporate B‐O species and lead to the formation of hollow BN spheres, in which the void size increases along with the increase of the treatment temperature.^[^ ^44^ ^]^

### 2.2 Degradability of Boron Oxynitride

**Figure 3:**
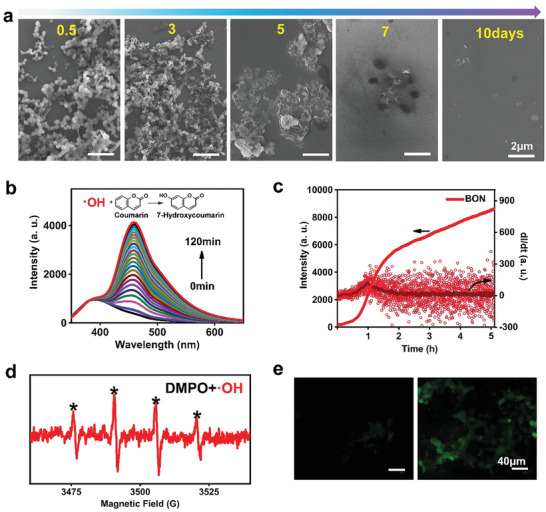
Enzymatic performance determinations for BON. a) SEM images of BON (0.1 mg mL^−1^) after degradations over different time at 37 °C. b) Fluorescence spectrum evolution for detection of ·OH radicals catalyzed by BON (200 µg mL^−1^) using coumarin as the trapping agent. Ex = 332 nm. c) Plots of fluorescence intensity (Em = 457 nm) and differential intensity versus reaction time. d) ESR spectra DMPO‐·OH spin adduct generated from BON‐catalytic decomposition of H_2_O_2_. e) DCFH‐DA fluorescence microscopic images of the 4T1 cells incubated without (left) and with (right) BON. The scale bars are 40 µm.

The biodegradability of BON nanozymes was investigated through observations of their structural evolutions in an aqueous solution. As shown in **Figure** **3**a, the morphology of the BON nanospheres changes continuously when incubated at a concentration of 0.1 mg mL^−1^ at 37 °C with shaking. First, the size of the nanospheres obviously shrinks in 0.5 day and then the structure of nanosphere changes into hollow spheres in 3 days. With continuing incubation, the original nanosphere‐like BON becomes a flat structure (in 5 days) and decomposes almost completely after 7 days’ degradation. Figure S4, Supporting Information, shows the XRD patterns of the decomposed products of BON, suggesting the formations of boric acid and NH_4_B_5_O_8_·4H_2_O after the degradation. These results suggest that the prepared BON nanozyme has excellent biodegradability, and would not cause long‐term toxicity when used as nanomedicines.

### 2.3 Peroxidase‐Like Catalytic Performances

To evaluate catalytic activity of BON and the efficiency of ·OH generations, a series of qualitative and semiquantitative analyses were performed. As shown in Figure 3b, the generation of ·OH radicals was first determined using the coumarin as a trapping agent to form the highly fluorescent 7‐hydroxycoumarin.^[^ ^45^ ^]^ After mixing the BON and coumarin solution with H_2_O_2_, the fluorescence peak at 457 nm arisen from 7‐hydroxycoumarin gradually increases. As shown in Figure 3c, the catalytic potency of BON is strong and endurable, which makes it an excellent nanozyme to continuously produce harmful ·OH. As a comparison, the typical Fe^2+^ solution shows strong but transitory catalytic activity for H_2_O_2_ decompositions (Figure S5, Supporting Information). However, there are no noticeable fluorescent signals of 7‐hydroxycoumarin detected at the same testing conditions for BON1000 and BON1400 samples, as shown in Figure S6, Supporting Information.

The generation of ·OH radicals was also more intuitively demonstrated by MB degeneration experiments and ESR detections. It was observed that after 1 h reaction of BON, H_2_O_2_, and MB, the absorbance of MB solutions decreased to 50% of the original value (Figure S7, Supporting Information). The MB was almost completely degraded after 12 h reaction (Figure S7, Supporting Information). We also carried out ESR measurement to detect the generated ·OH radicals using DMPO as a spin‐trapping agent. After catalytic reaction for 20 min, the ESR spectrum confirms the spin signals of DMPO‐trapped ·OH radicals with the ratio of 1:2:2:1 (Figure 3d).^[^ ^46^ ^]^ Furthermore, the intracellular ROS generation was confirmed by a DCF‐DA assay. As shown in Figure 3e, prominent green fluorescence appears in 4T1 cells that are incubated with the BON nanozyme and treated with the DCF‐DA agent, suggesting the efficient ROS accumulations in live 4T1 cells. These results verify the extracellular and intracellular peroxidase‐like behaviors of the developed BON nanospheres.

The ·OH radicals react with the DNA, enzymes and other biomolecules in the cell, causing damages and cell apoptosis. On one hand, the tumor shows increased ROS levels compared with normal tissues.^[^ ^47^ ^]^ The accumulation of ROS in cancer cells makes the cells under a high oxidative stress state, and may cause the destructions of cell components and lead to cell apoptosis or necrosis.^[^ ^48^ ^]^ On the other hand, tumor tissues are prone to higher sensitivity to ROS rather than the normal ones because of the lower level of antioxidant enzymes in tumors.^[^ ^49^ ^]^ Therefore, intracellular stimulation of forming high levels of ROS becomes a promising anti‐cancer strategy.^[^ ^50^ ^]^ ROS induces cell apoptosis through the death receptor‐dependent extrinsic pathway including tumor necrosis factor‐alpha and Fas ligand and the mitochondrial intrinsic pathway.^[^ ^51^ ^]^ High concentration of ROS can induce opening of mitochondrial permeability transition pores through regulating the conformation of adenine nucleotide translocase in mitochondrial inner membrane, and further reduce mitochondrial transmembrane potential, release cytochrome‐c, and apoptosis‐inducing factor, and then activate caspase signaling cascade and induce apoptosis.^[^ ^52^ ^]^

### 2.4 Biocompatibility and Cellular Uptake

**Figure 4:**
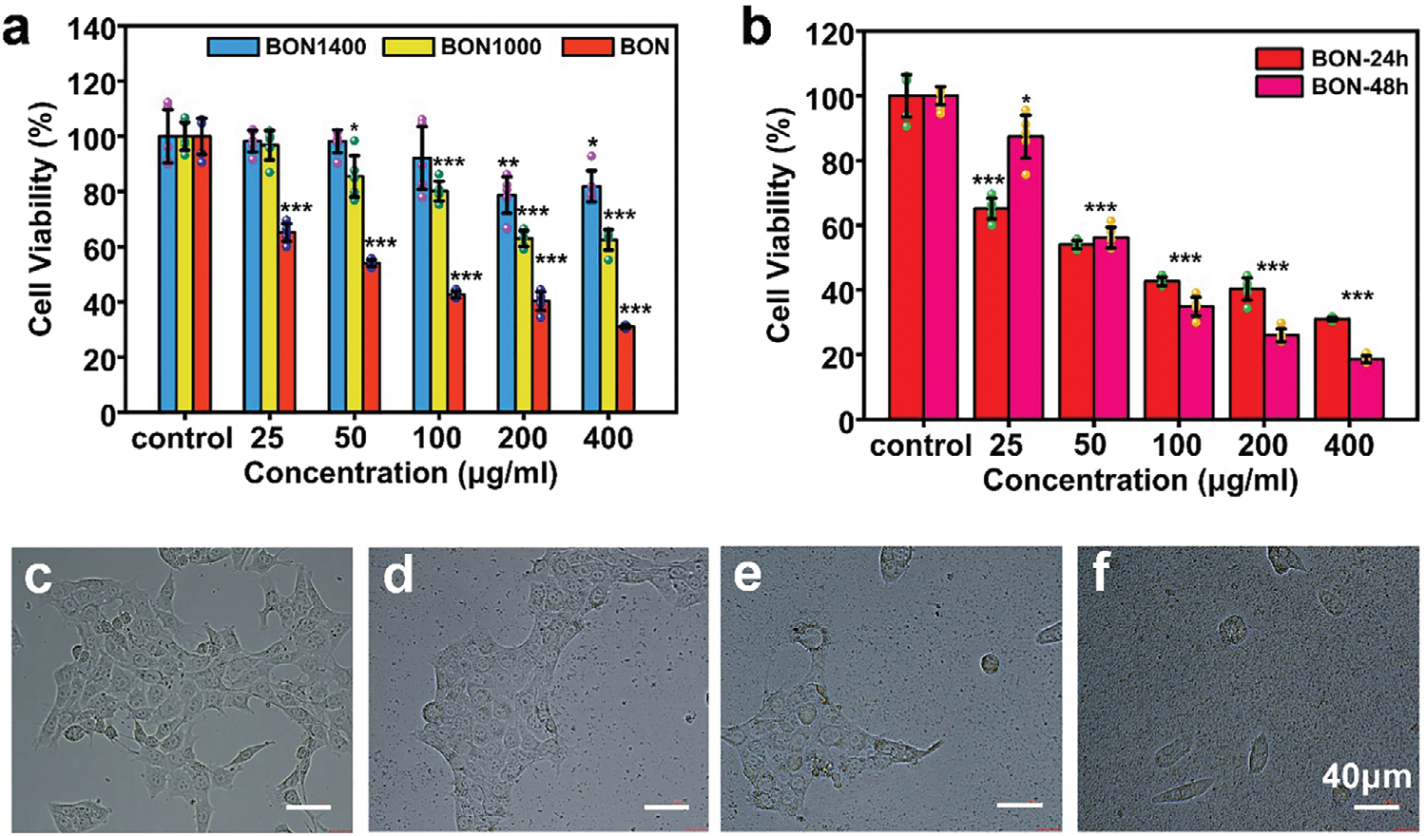
In vitro viability assays of BON nanozyme. a) Viability of 4T1 cells incubated with BON, BON1000, and BON1400 at different concentrations for 24 h. b) Cell viability incubated with BON for 24 and 48 h. c–f) Optical microphotographs of 4T1 cells cocultured with BON at different concentrations (c: control, d: 25 µg mL^−1^, e: 100 µg mL^−1^, f: 400 µg mL^−1^) for 24 h. The scale bars in (c–f) are 40 µm. Data in (a,b) are shown as mean ± SD; *n* = 5 per group. Student's *t*‐test was used to calculate *p*‐values by SPSS software. *p < 0.05, **p < 0.01, ***p < 0.001.

Since the demonstrations of high ·OH production efficiency for the BON nanozyme, it is of great interest to know the inhibition effect for cancer cells. **Figure** **4**a illustrates the viability of 4T1 cells cocultured with BON, BON1000, and BON1400 at different concentrations for 24 h. At the same concentration, the cytotoxicity of BON is much higher than those of BON1000 and BON1400, suggesting a remarkable inhibition effect of the BON for 4T1 cells. The regarded cell viability is 50% at the BON concentration of 50 µg mL^−1^ and further reduces to 31% when the concentration increases to 400 µg mL^−1^. In contrast, when cocultured with BON1000 and BON1400, even at a high concentration of 400 µg mL^−1^, the resultant cell viabilities still reach to 63% and 82%. The cytotoxicity of BON1400 is not significantly different from that of pure *h*‐BN (Figure S9, Supporting Information), which has been verified to have good biocompatibility. After coculturing with BON for 48 h, the cell viability is further reduced to 18% (Figure 4b), indicating the high potency for in vitro 4T1 cell inhibitions. From the microscopic images (Figure 4c–f), it can also be seen that the cell density tends to reduce and the cell morphology utterly deteriorates along with the increase of the BON concentration.

Considering the accumulation effects of the BON nanospheres in the reticuloendothelial system, practically in liver,^[^ ^37^ ^]^ we further carried out in vivo experiments and performed serum biochemical analysis and H&E staining experiments of the Balb/c mice administered with BON samples after 7 days. As shown in Figure S10, Supporting Information, there are no significant effects on mouse liver functions compared with control group, suggesting good biocompatibility of BON nanozyme for normal tissues.

**Figure 5:**
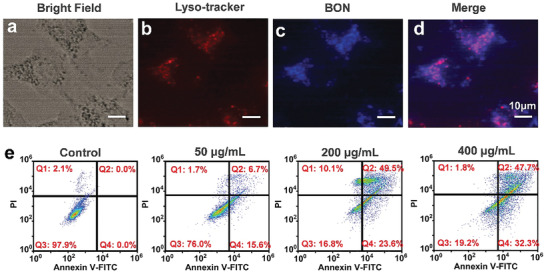
Cellular uptake and cell death mechanism assays. a) Bright‐field microscopic image of 4T1 cells that incubated with BON and Lyso‐Tracker Red. Fluorescence microscopic images of 4T1 cells at the b) Ex = 540 nm and c) Ex = 350 nm. d) Merged image of (b) and (c). e) Flow cytometry with Annexin V‐FITC/PI assay of 4T1 cells after 24 h co‐incubation with BON at different concentrations. The scale bars in (a–d) are 10 µm.

The BON nanospheres are blue fluorescent under UV light excitation (Figure S12, Supporting Information), which can be employed for material tracking. As revealed by colocalization assay (**Figure** **5**a–d), the BON nanozymes are prone to accumulate at the 4T1 cell lysosomes. It is also of interest to understand cell uptake mechanisms for nanomedicines. Previous studies have revealed an important role of particle size for a nanomaterial to enter cells. Nanomaterials with diameters larger than 500 nm tend to enter cells through a caveolae‐mediated internalization mechanism, while the ones with a diameter less than 200 nm tend to enter cells through clathrin‐mediated endocytic pathways.^[^ ^53^ ^]^ The diameters of the prepared BON nanospheres are mainly located within a range of 100–500 nm, and thus are assumed to enter cells through an endocytic pathway mediated by caveolin or clathrin.^[^ ^54^ ^]^ BON can be accumulated in lysosomes and behave like an artificial peroxidase, thereby increasing the level of ROS.

Since both the generated ROS and released B compounds can reduce the 4T1 viability,^[^ ^37^, ^55^ ^]^ it is necessary to distinguish the impacts between these effects. We performed dialysis for the BON solutions and collected the eluates for cytotoxicity assay. As shown in Figure S14, Supporting Information, the released B compound from BON has mild cytotoxicity for 4T1 cells after co‐incubation at the same condition. It exhibits no obvious cytotoxicity for the eluates from the BONs with concentrations lower than 100 µg mL^−1^; when the BON concentrations are 200 and 400 µg mL^−1^, the measured cell viabilities are 85% and 70%, respectively, which are much higher than those directly treated by the BON samples. These results suggest that the cytotoxicity of BON for 4T1 cells should mainly originate from the generated ROS catalyzed by peroxidase‐mimetic nanozyme. This is very different from the BON1000 sample, whose dialysis eluate shows very close cytotoxicity results with those directly treated by BON1000, indicating a B release‐dominated cytotoxicity origin of the sample. This is in accordance with the previous report.^[^ ^37^ ^]^ The present studies indicate that, besides the cytotoxicity arising from the released soluble B compounds in the BON1000, the cytotoxicity for the BON nanozyme mainly roots in the peroxidase‐mimetic formation of ·OH radicals, a mechanism seldom reported for the known BN materials.

In recent years, there have been numerous cutting‐edge catalysts developed for industrial reactions based on oxygen modified *h*‐BN structures. For example, the edge O‐terminated BN nanosheets were found to have highly selective catalytic dehydrogenation of propane to propene.^[^ ^56^ ^]^ BN nanosheet‐constituted microspheres were developed for catalytic oxidative dehydrogenation of propane to produce propylene and ethylene.^[^ ^57^ ^]^ And indeed, the property of catalytic generations of ·OH radicals had been reported for BN quantum dots, which were prepared by sonication and hydrothermal treatment of edge hydroxylated BN nanosheets. It was proposed that the free B radicals at the edges and defective sites of the BN quantum dots were responsible for the resultant catalytic performances because of the detected quenching effect of DPPH.^[^ ^58^ ^]^ This result highlights the potentials of BN nanomaterials for artificial nanozyme applications. As the structural analog of *h*‐BN, carbon nanomaterials doped with nitrogen atoms have been reported to show enzyme‐like activities.^[^ ^28^ ^]^ The regarded peroxidase‐like behaviors therein were ascribed to the involvement of N‐O structures.^[^ ^28^ ^]^ In the present study, rich N‐O bonds were found in the BON sample, which completely disappeared in the samples after annealing at 1000 and 1400 °C, and thus were also tentatively attributed as the catalytic sites. Successful clarifying the catalytic mechanisms will unambiguously guide future rational designs of high‐performance artificial nanozymes.

### 2.5 Cell Death Mechanism Induced by Boron Oxynitride Nanozyme

Flow cytometry was adopted to explore the anticancer mechanism of BON nanozyme with Annexin V‐FITC/PI assay. Annexin V‐FITC+/PI− stained cells indicate early apoptotic, while the cells stained Annexin V‐FITC+/PI+ are considered as late stage apoptotic. The Annexin V‐FITC−/PI+ staining result accounts for cell necrotic. As shown in Figure 5e, the BON nanozyme exhibits dose‐dependent cytotoxicity. With the increase of BON concentration, the proportion of early apoptosis and late apoptosis of 4T1 cells increases significantly. When the concentration of BON reaches 200 and 400 µg mL^−1^, 73.1% or 80.0% of total cells are in early and late apoptosis, respectively, accompanied with only 10.1% and 1.8% belonging to cellular necrosis (similar to the control group, 2.1%); the proportions of early and late apoptotic cells in the BON samples with 200 and 400 µg mL^−1^ are about 3.3 and 3.6 times as high as that for the concentration of 50 µg mL^−1^; there are no early or late apoptosis cells detected in the control group. The dialysate of BON (400 µg mL^−1^) was also collected to coculture with 4T1 cells; the flow cytometry in Figure S15, Supporting Information, reveals 6.1% of the cells in early apoptosis and 4.0% in late apoptosis, the numbers which are significantly lower than those for the samples incubated with BON directly at the same concentration. This also supports our claim that the peroxidase‐like catalytic generation of ROS of BON nanozyme plays the key role in regulating the apoptosis of 4T1 cells.

### 2.6 In Vivo Anticancer Effect

**Figure 6:**
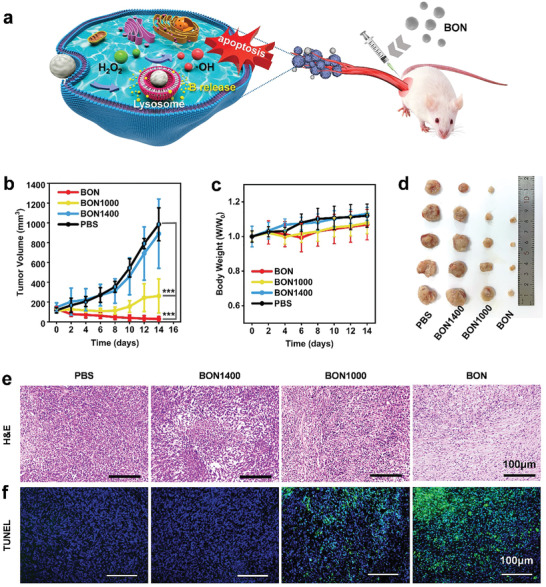
In vivo evaluations of BON nanozyme for breast cancer therapy. a) Schematic illustration of therapeutic mechanism of BON. b) Tumor growth curves of 4T1 tumors in BALB/c mice treated at different conditions. c) Body weights of different groups of mice. d) Photographs of exfoliated tumors of different groups. e) Optical photographs of tumor sections stained with H&E. f) Corresponding fluorescent images of tumor sections subjected to TUNEL staining. The scale bars in (e,f) are 100 µm. Data in (b,c) are shown as mean ± SD; *n* = 5 per group. Student's *t*‐test was used to calculate *p*‐values by SPSS software. *p < 0.05, **p < 0.01, ***p < 0.001.

To further test and understand the anticancer effect of BON nanozyme in vivo, we explored the therapeutic effect of BON on mice bearing 4T1 tumors (**Figure** **6**a). When the subcutaneously formed tumors grew up to ≈100 mm^3^, the mice were subjected to the injections of samples as well as PBS as a control. Among all experimental groups, BON significantly suppresses the tumor growth with the average tumor volume of 29 mm^3^ after 14‐day treatment (Figure 6b). For the BON1000 group, it was found that the growth rate of tumors was remarkably reduced compared with that of the control group. And the tumor growth for the inert BON1400‐treated mice shows no significant difference with that of the control. After 14‐day treatment, the average tumor volumes in the control group, BON1400, and BON1000 are 985, 891, and 261 mm^3^, respectively. These results also confirm that the BON1000 can suppress tumor growth by about 74% compared with the control because of releasing B compounds.^[^ ^37^ ^]^ While to the BON nanozyme, besides the release of B species, the catalytic generations of ROS lead to excellent anticancer potency in vivo, which achieves the inhibition of tumor growth by 97%. And amazingly, the tumor tissue in one mouse of BON group was found to be completely eliminated through visual inspection (Figure 6d). During the treatment, there was no significant difference in the mice body weight of the experimental groups compared with the control, indicating a high biocompatibility of BON samples for biomedical applications (Figure 6c).

Thereafter, the tumor tissues were stained by H&E and triphosphate nick end labeling (TUNEL) to evaluate the therapeutic effects in different groups. From the results of H&E staining (Figure 6e), the apoptosis/necrosis of the tumor tissue in the BON group is the most serious, which is consistent with the results of the tumor growth curve. In addition, from the results of H&E staining of the main organ tissues (heart, liver, spleen, lung, and kidney) of each group of mice (Figure S16, Supporting Information), there are no obvious damages for these organ tissues, which further proves the excellent biosafety of the BON nanozyme. The apoptotic cells on the TUNEL stained tissue sections show green color fluorescence, and the nuclei exhibit blue fluorescence. From Figure 6f, it is clear that the BON‐treated tumors have the strongest green fluorescence among all the groups, indicating that the BON can effectively induce apoptosis for the 4T1 cells and function as a potent anticancer drug.

The development of nanomedicine provides tremendous opportunity for cancer diagnosis and therapy, which can maximize the clinical efficacy and reduce side effects compared with traditional medicine forms.^[^ ^59^ ^]^ In recent years, the potentials of BN nanomaterials for anticancer and biomedicine applications start to be recognized. First, as a type of high B‐rich agent, BNNTs were proposed for neutron capture cancer therapy.^[^ ^60^ ^]^ Boron neutron capture therapy requests a high B content and targeted delivery capability of the neutron capture agent to increase the therapeutic efficacy relative to conventional radiotherapeutic drugs. Numerous B‐rich nanoparticles have been developed with remarkable B species enrichment in tumors compared with blood and other organs, such as, BNNTs,^[^ ^61^ ^]^ boron carbide,^[^ ^62^ ^]^ and carborane cage‐attached CNTs.^[^ ^63^ ^]^ Through morphological controls and surface modifications, BN nanomaterials could be used as an effective carrier of doxorubicin and realized much enhanced intracellular drug delivery to LNCap prostate cancer cells,^[^ ^35^ ^]^ or load Auristatin PE to trigger the mitochondrial‐mediated apoptosis of Hep G2 cells.^[^ ^64^ ^]^ Surface chemical modifications of BN nanomaterials could further enhance the specific targeting performances by ligand‐receptor recognitions and promote the therapeutic efficacy.^[^ ^43^, ^65^ ^]^ Recent advances suggested that hollow BN nanospheres could slowly and endurably release water‐soluble B compounds, which were found to efficiently inhibit LNCap prostate cancer cell growth. This result represents a remarkable progress of BN nanomedicine toward non‐invasive therapy of cancers.^[^ ^37^ ^]^ However, compared with other nanomedicines, designs and explorations of BN nanomaterials on anticancer applications are still in their infancy. This study uncovers the peroxidase‐like nanozyme activity of BON nanospheres, providing a new powerful tool based on BN structures toward potent cancer therapies.

One critical issue in cancer treatment is the difficulty eliminating cancer cells completely, which will cause failures of the therapies. To eliminate cancer cells as much as possible, an emerging strategy is to combine different diagnostic and therapeutic functions within one material platform. For example, by integrating multiple functions of photothermal, photodynamic, magnetotherapy, chemotherapy together in carbon materials,^[^ ^66^ ^]^ or up‐conversion nanoparticles,^[^ ^67^ ^]^ the reported cancer therapeutic efficacies were enormously improved compared with the corresponding monofunctional materials. Herein, for the BN nanomaterial, it is possible to in parallel integrate the therapeutic functions of neutron capture therapy, boron‐releasing therapy, chemotherapy, as well as, the nanozyme therapy that discovered in the present work. In addition, BON nanozyme exhibits strong blue fluorescence, which can be exploited for real‐time tracking of the treated materials. This is also readily integrable into the BN nanomedicine platform. It is known that the bandgap of *h*‐BN could be effectively tuned through heteroatom‐doping or functionalization. Through oxygen doping and hydroxyl functionalization, the bandgap of BN was successfully reduced to 2.1 eV and the resultant BN materials showed strong cyan fluorescence.^[^ ^39^ ^]^ All these progresses highlight the great potentials to design and develop BN nanomedicine platforms integrated with multiple different diagnostic and therapeutic functions.

## 3 Conclusions

In summary, we have designed the biodegradable peroxidase‐mimetic BON nanospheres containing rich N‐O bonds for efficient breast cancer therapy. In vitro experiments have shown that BON can effectively and continuously catalyze hydrogen peroxide to generate highly reactive hydroxyl radicals. Colocalization experiment has revealed that the blue‐fluorescence BON nanozyme can accumulate specifically in lysosomes and stimulate 4T1 cells to generate hydroxyl radicals, thereby inducing cell apoptosis. The BON nanozyme has been found to reduce the viability of the 4T1 cancer cells in vitro by 82% in 48 h. It also exhibits high potency for breast tumor inhibitions by 97% after 14‐day treatment, much more effective than the inert and B‐releasing BN nanospheres. The present study integrates multiple functions of peroxidase‐like nanozyme, boron‐release therapy, and drug tracking into the biodegradable BON nanomaterial, opening the way toward rational designs of the multi‐mode BN nanomedicine platform for potent cancer diagnosis and therapies.

## 4 Experimental Section

### Synthesis of Boron Oxynitride

The BON sample was synthesized through a pyrolysis method.^[^ ^44^ ^]^ As shown in Figure S1, Supporting Information, trimethyl borate vapor was carried by N_2_ and NH_3_ gas (N_2_: 800 mL min^−1^, NH_3_: 300 mL min^−1^) and introduced into a horizontal tube furnace with a temperature of 1000 °C. White solid product formed and was collected at the outlet tube wall regions of the quartz tube. For comparison, the BON product was proceeded for further heat treatments at 1000 and 1400 °C for 4 h to generate BON1000 and BON1400 samples, respectively.

### Material Characterizations

The morphology and composition of samples were characterized by a SEM (TESCAN MIRA3 LMH) and a Cs‐correction TEM miscroscope equipped with EELS (Titan Themics G2 60–300). Surface zeta‐potential and particle size were determined using a Zetasizer Nano ZSP (Malvern Instruments) based on DLS. XRD were measured on a BrukerAXS D8 Advance using Cu K*α* target. XANES data was collected on the XAFCA beamline at Singapore synchrotron light source using a transmission mode. The electron energy was 700 MeV and the beam current was below 200 mA. FTIR were taken on a Bruker Vertex 70 FTIR spectrophotometer in the range of 4000–400 cm^−1^. XPS (Thermo Scientific K‐Alpha) was employed to study the chemical states of samples. All the XPS spectra were calibrated by principal C1s binding energy peak at 284.6 eV. ESR spectra were collected on a EMXmicro‐6/1/P/L system. Photoluminescence (PL) spectra were acquired on a JASCO FP‐8500 spectrofluorometer, and UV–vis spectra were collected on a SHIMADZU UV‐2600 spectrophotometer. Fluorescence images were taken by an inverted fluorescence microscope (Motic, MXH‐100).

### Evaluation of ·OH Generation

First, to qualitatively determine generated hydroxyl radicals (·OH), 500 µL 2 mg mL^−1^ BON solution was mixed with 20 µL H_2_O_2_ and a spin‐trapping agent (DMPO, 0.1 m) in a dark environment. After 20 min, the solution was transferred to a quartz tube for ESR measurement. Besides, the catalytic generation of ·OH was also evaluated by the degradation of methylene blue (MB). Specifically, 100 µL 12.5 mg L^−1^ MB solution was added into a 96‐well plate. Then, 100 µL 200 µg mL^−1^ BON and different amounts of H_2_O_2_ were added to make the final H_2_O_2_ concentrations of 10, 1, and 0.1 mm, respectively. The absorbance at 620 nm was measured by a microplate reader (Thermo Scientific, Multiscan FC). For semiquantitative evaluations, coumarin was used as the trapping agent of ·OH to form the highly fluorescent 7‐hydroxycoumarin to assess the produced ·OH.^[^ ^45^ ^]^ Specifically, a 2 mL solution with 200 µg mL^−1^ BON and 5 mm coumarin was prepared. Then, 5 µL H_2_O_2_ was added to the solution to initiate the reaction. The fluorescence intensity of 7‐hydroxycoumarin (Ex: 332 nm Em: 467 nm) was measured in every 5 min.

### Intracellular Reactive Oxygen Species Detection

4T1 cells were sowed on a 96‐well plate with the density of ≈1 × 10^4^ cells per well, which were cultured in 90 µL Dulbecco's modified Eagle's medium (DMEM, Gibco) containing 10% fetal bovine serum (Gibco), 1% antibiotic, and antimycotic solution (Gibco) in a humidified atmosphere with 5% CO_2_ at 37 °C. After the complete attachment, 10 µL BON (4 mg mL^−1^) was added and incubated for 6 h and then the culture medium was replaced by the fresh one containing 5 µM DCF‐DA. After 10 min, the cells were washed with DMEM three times to remove the adsorbed DCF‐DA on the cell surfaces and observed by an inverted fluorescence microscope under the excitation wavelength of 480 nm.

### In Vitro Cell Viability Assay

4T1 cells were seeded and cultured in a 96‐well plate at the same conditions as described above. Then, 10 µL BON, BON1000, and BON1400 solutions were added to the culture medium to make the final sample concentrations of 25, 50, 100, 200 and 400 µg mL^−1^, respectively. After coculturing for 24 h, cell viabilities were measured by cell‐counting kit‐8. The optical density (OD) was recorded on the microplate reader with a filter of 450 nm. By comparing the absorbance between the control group and the experimental groups, the cell viability was calculated.

### Colocalization Assay

BON sample was cocultured with cells for 4 h. Then, the cells were washed with PBS to remove the attached samples on cell surfaces. Then Lyso‐Tracker Red (Lyso‐Tracker Red, *λ*_Ex_ = 500–577 nm, *λ*_Em_ = 590 nm, Beyotime) was added for co‐incubation with cells for additional 10 min. Finally, the fluorescence images of the cells were recorded by an inverted fluorescence microscope under the required excitation wavelengths.

### Apoptosis Assay

The Annexin V‐FITC apoptosis detection kit was used to analyze the 4T1 cell apoptosis exposed to BON, BON1000, and BON1400 by flow cytometry. In brief, 3 × 10^6^ 4T1 cells were sowed in a six‐well plate. After 24 h incubation, the nanozyme samples were added to make the final concentrations of 50, 200, and 400 µg mL^−1^, respectively. The cells were cocultured for another 24 h and washed with PBS. Then, the Annexin V‐FITC binding buffer was added to prepare the cell suspension. Finally, 5 µL of Annexin V‐FITC and 10 µL of PI solutions were added and incubated at room temperature in dark for 15 min for flow cytometry analysis (BD FACSCalibur).

### In Vivo Anti‐Tumor Experiments

All animal experiments were carried out according to Hunan University's guidelines on animal use and care. The tumor models were established by injecting 100 µL 4T1 cell suspension (around 7 × 10^6^ cells) subcutaneously into BALB/c mice (≈16 g, female). After raising for 7 days, the tumor volumes in mice grew to ≈100 mm^3^. Then, 20 BALB/c mice were divided into four groups randomly (*n* = 5). They were injected with BON, BON1000, and BON1400 solutions intratumorally. Every mouse was subjected to an injected dose of 0.4 mg on day zero and day seven. While in the control group, PBS was injected into tumors instead. In order to evaluate the effect of anti‐tumor treatment, the body weights and tumor volumes of mice were recorded every other day. After 14 days treatment, mice were euthanized to make their neck dislocated painlessly. Then, tissues from heart, liver, spleen, lung, kidney, and mouse tumors were collected and stained with hematoxylin and eosin (H&E) and transferase‐mediated deoxyuridine TUNEL staining to evaluate the biological safety and antitumor effects.

### Statistical Analysis

All the data were presented as mean ± SD. Student's *t*‐test and one‐way ANOVA method were adopted to assess the difference between groups by using SPSS software. Differences were considered statistically significant at *p* < 0.05. (*p < 0.05, **p < 0.01, ***p < 0.001)

## Conflict of Interest

The authors declare no conflict of interest.

## Author Contributions

Q.W. conceived the research and supervised the project. L.Z. performed the material synthesis, characterization, and anticancer assays. Y.H., Z.C., and K.J. participated in material preparations and characterizations. All the authors were involved in data analysis and result discussions. L.Z. and Q.W. wrote the manuscript with the inputs from all authors.

## Supporting information

Supporting Information

## Data Availability

The data that support the findings of this study are available from the corresponding author upon reasonable request.
