# Supplementary material for: Biodegradable and Peroxidase‐Mimetic Boron Oxynitride Nanozyme for Breast Cancer Therapy
Source: Adv Sci (Weinh). 2021 Jun 30;8(16):2101184. doi: 10.1002/advs.202101184 (PMC8373162; doi:10.1002/advs.202101184)
Supplement: Supplementary file 1 — Supporting Information [file ADVS-8-2101184-s001.pdf]

WILEY-VCH

© 2021 Wiley-VCH GmbH

ADVANCED SCIENCE
Open Access

Supporting Information

for *Adv. Sci.*, DOI: 10.1002/advs.202101184

Biodegradable and Peroxidase-Mimetic Boron Oxynitride Nanozyme for Breast Cancer Therapy

*Lula Zeng, Yuxin Han, Zhiwei Chen, Kang Jiang, Dmitri Golberg, Qunhong Weng**

Supporting Information

Biodegradable and Peroxidase-Mimetic Boron Oxynitride Nanozyme for Breast Cancer Therapy

*Lula Zeng, Yuxin Han, Zhiwei Chen, Kang Jiang, Dmitri Golberg, Qunhong Weng**

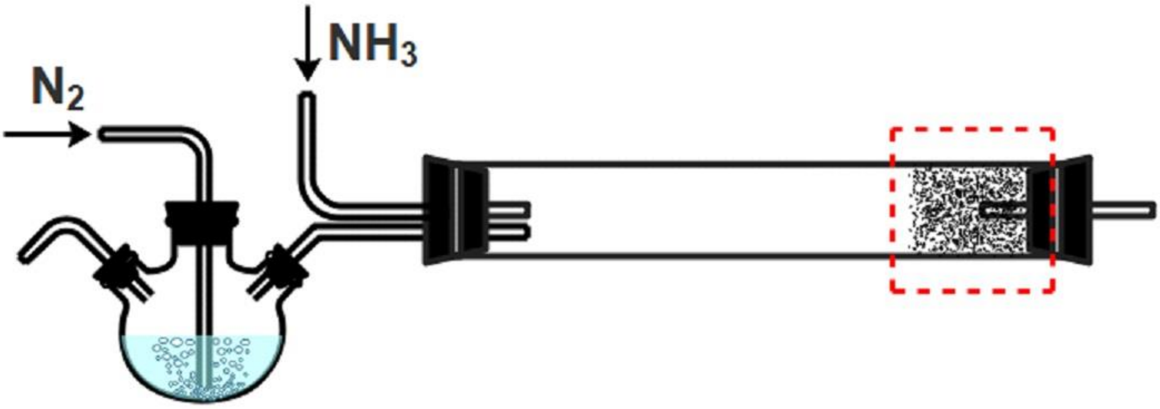

Figure S1. Schematic diagram of the synthetic procedure.

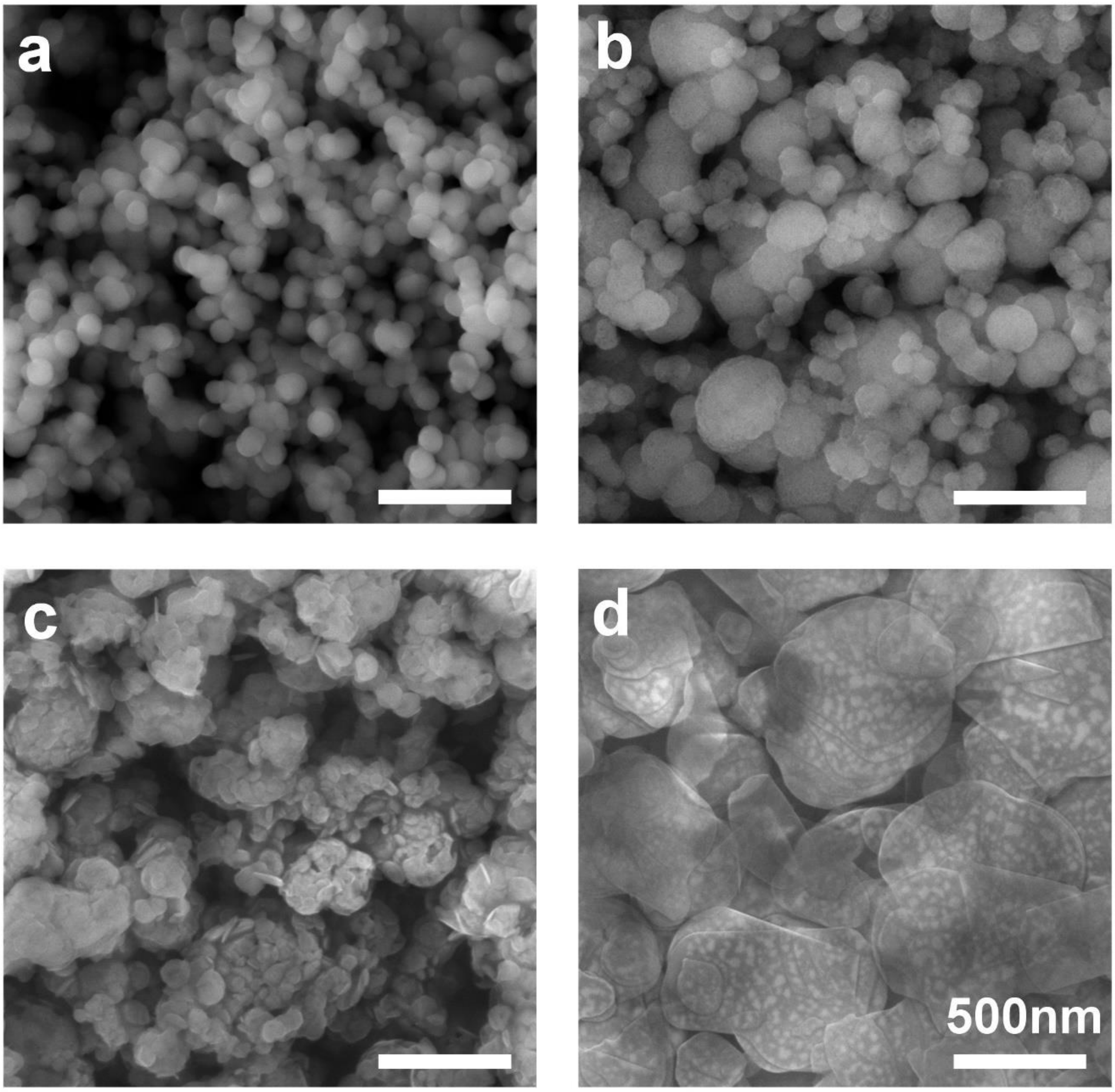

Figure S2. (a, b, c, d) SEM images of BON, BON1000, BON1400, and commercial *h*-BN. The scale bars are 500 nm.

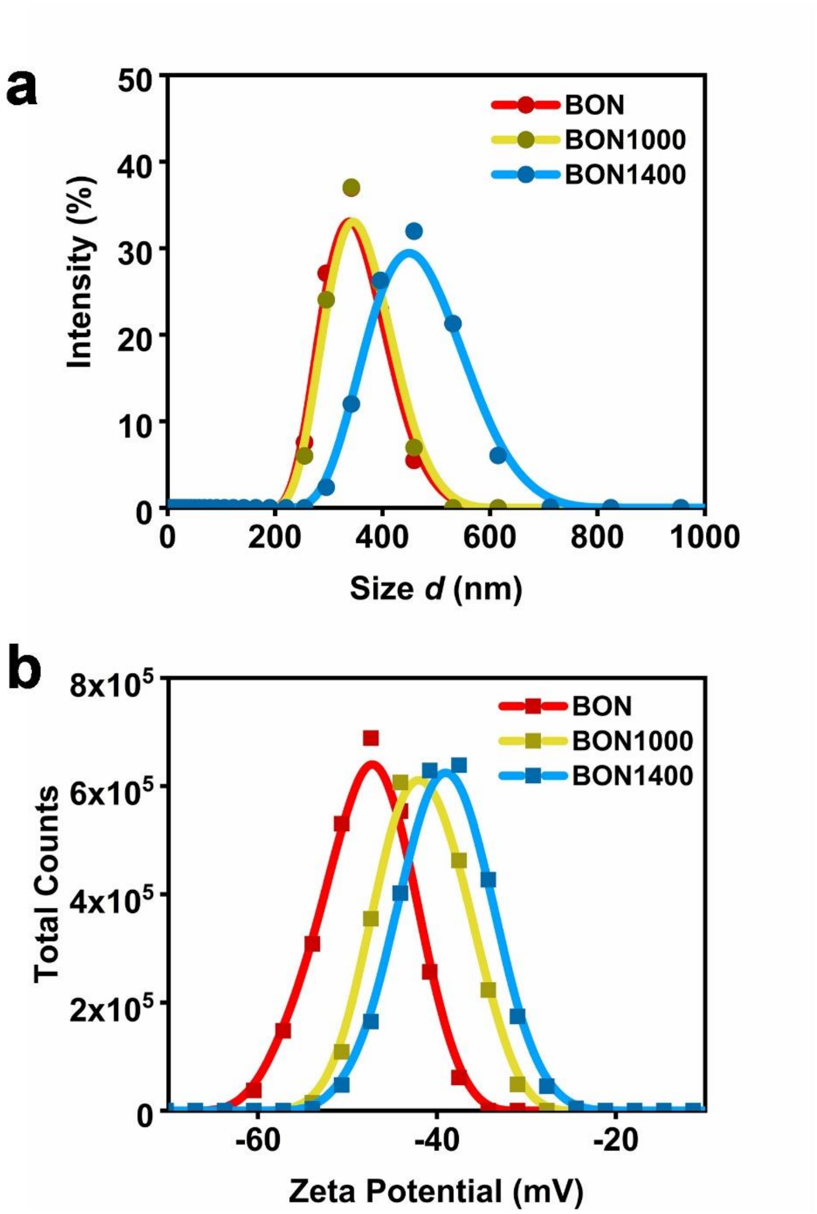

Figure S3. (a) DLS analyses of BON, BON1000, and BON1400. (b) Zeta potential of BON, BON1000, and BON1400 in deionized water.

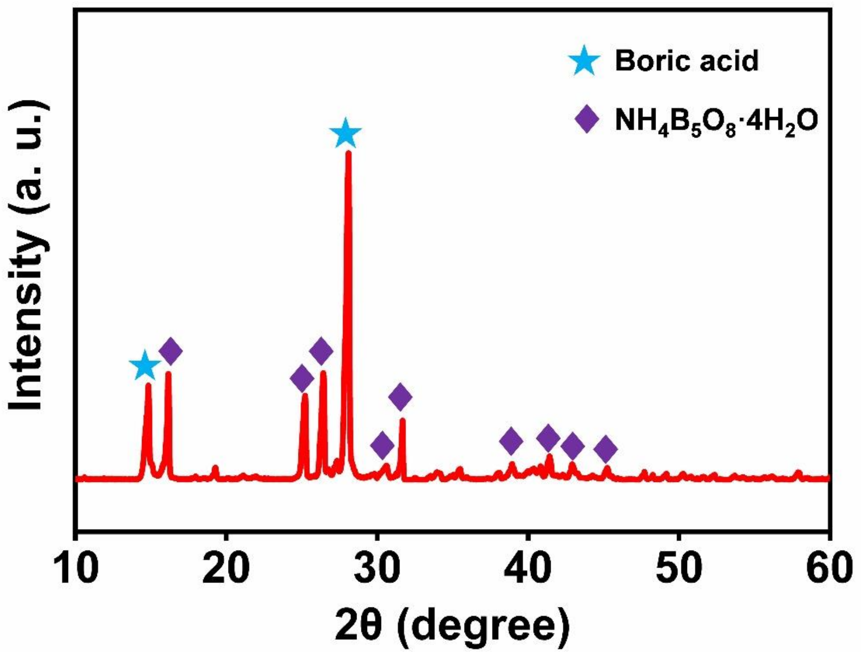

Figure S4. XRD pattern of degradation products of BON.

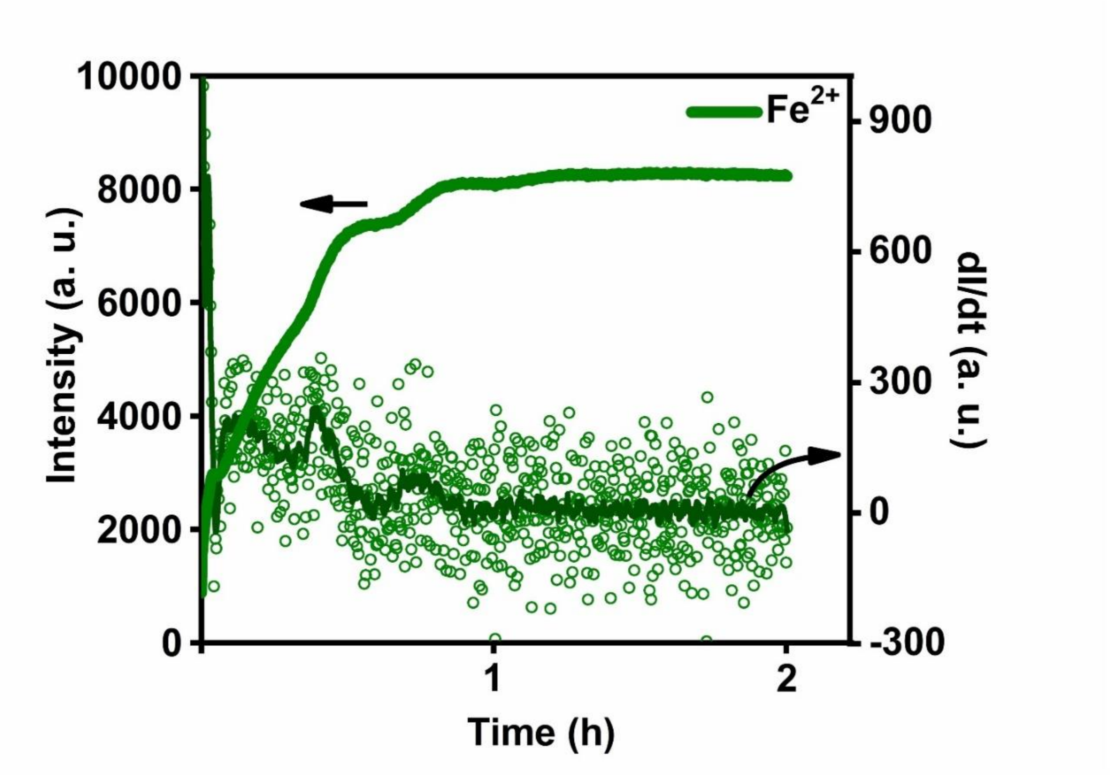

Figure S5. Plots of fluorescence intensity (*Em* = 457 nm) and differential intensity of Fe^{2+} versus reaction time.

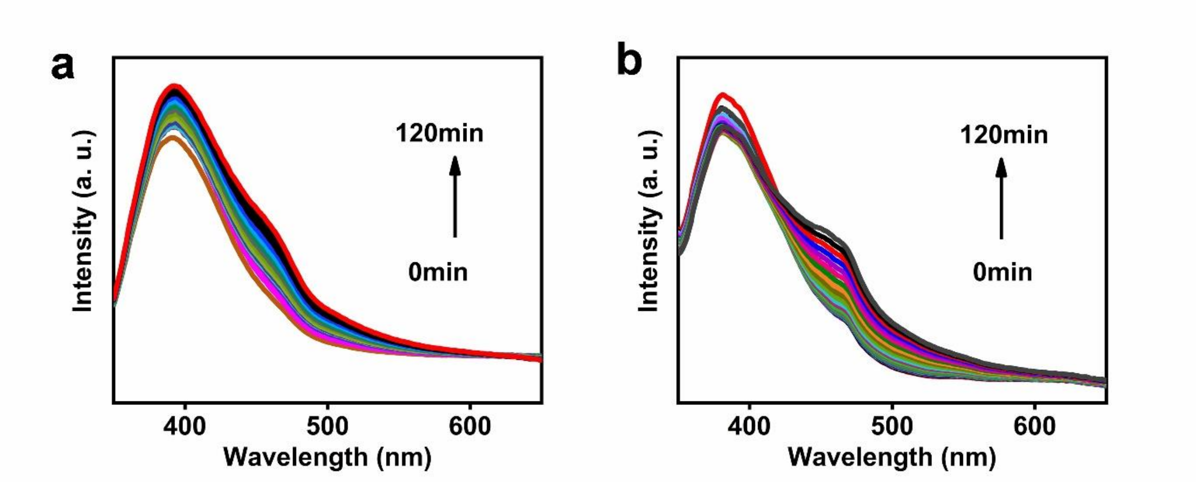

Figure S6. Fluorescence spectra evolution of coumarin solution used as the trapping agent for detection of ·OH radicals catalyzed by BON1000 (a) and BON1400 (b) (200 μg/ml). E_x = 332 nm. With the increase of reaction time, fluorescence emissions at 457 nm were not emerging significantly in the samples of BON1000 and BON1400, which indicated that the BON1000 and BON1400 could not catalyze hydrogen peroxide to produce hydroxyl radicals, and thus not exhibited the peroxidase-like performances.

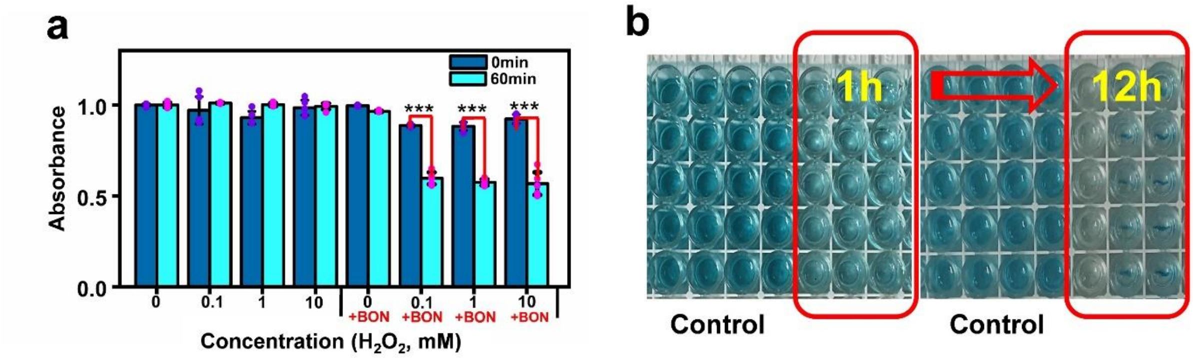

Figure S7. (a) Absorbance of MB solution degenerated by BON and different-concentration H_2O_2 for 1 h. The detection wavelength for MB is 620 nm. Data are shown as mean ± SD; n=5 per group. Student's t-test was used to calculate p-values by SPSS software. *p<0.05, **p<0.01, ***p<0.001. (b) Photographs of MB solutions treated by BON and H_2O_2 in a 96-well plate after 1 hr and 12 hrs.

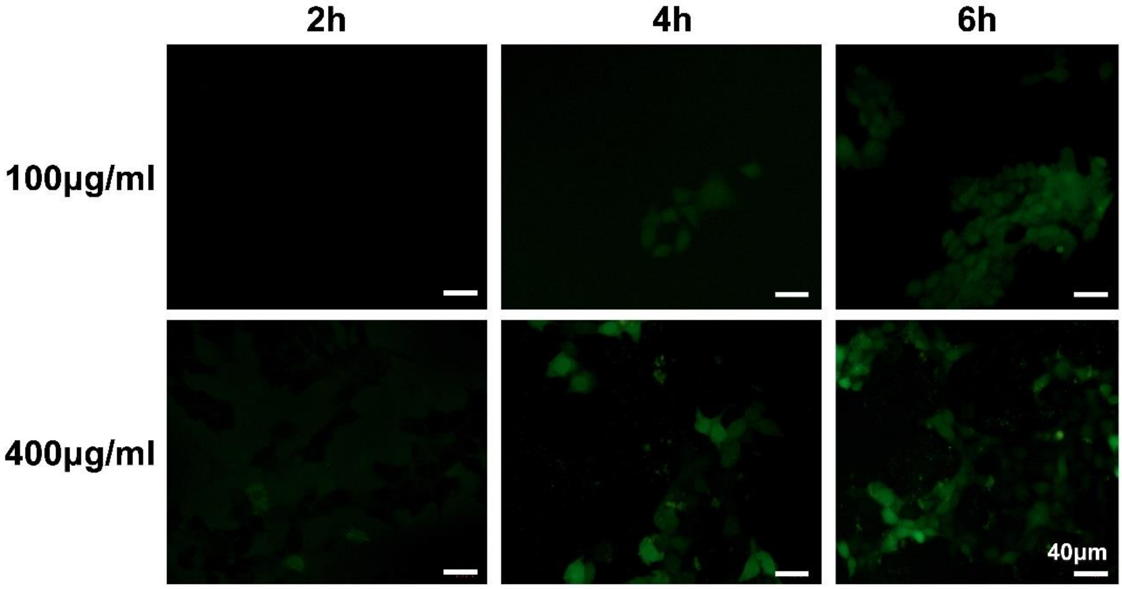

Figure S8. DCFH-DA fluorescence microscopic images of the 4T1 cells incubated with BON with different concentration and time. After incubating Fe^{2+} ions with cells, adding DCF-DA, green fluorescence can be detected in the cells, indicating that ROS is produced in them.

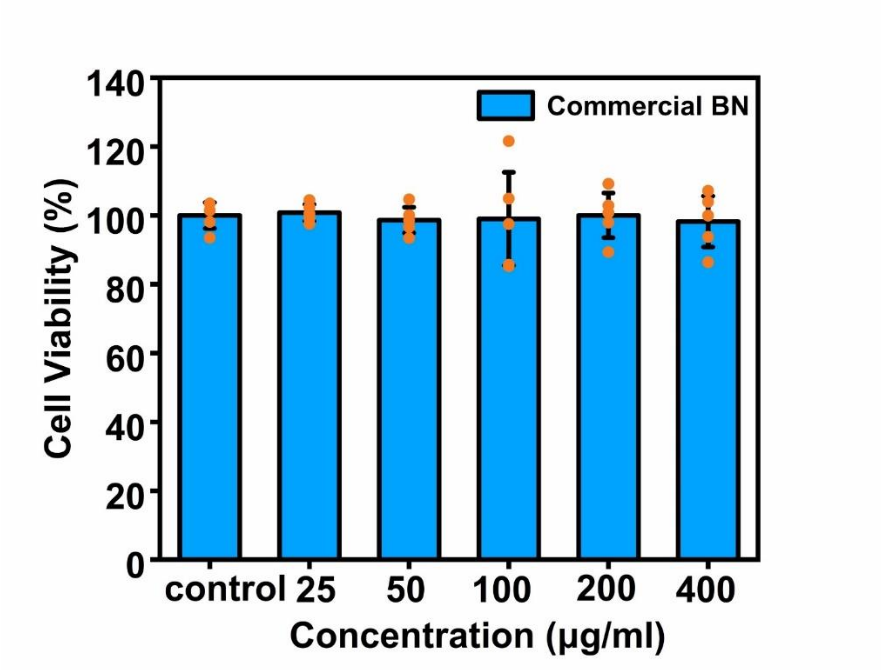

Figure S9. Cytotoxicity of commercial *h*-BN. Data are shown as mean±s.d. With an increase of commercial *h*-BN concentration, there is no significant difference in cell survival rate compared with the control group, which indicates that the commercial *h*-BN has good biocompatibility. Data are shown as mean ± SD; n=5 per group. Student's t-test was used to calculate p-values by SPSS software. *p<0.05, **p<0.01, ***p<0.001.

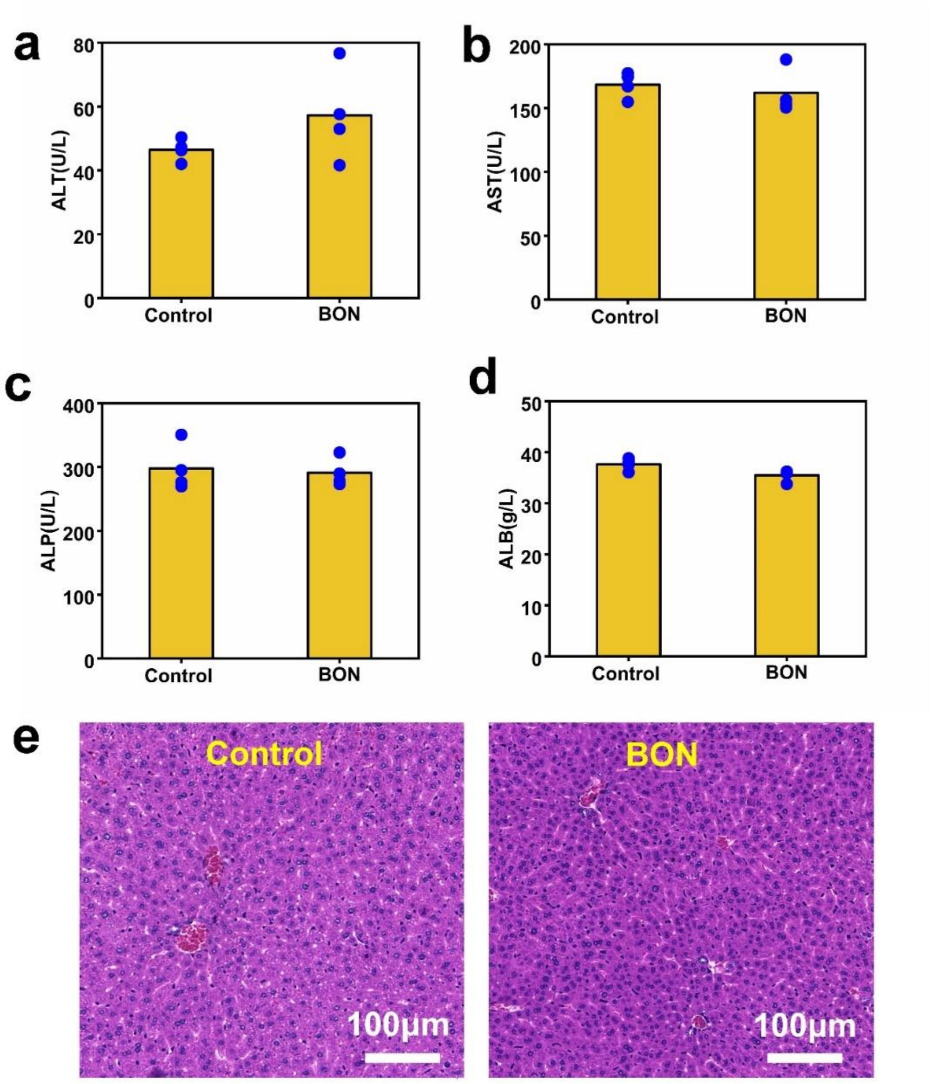

Figure S10. Evaluation of liver injury caused by intravenous injection of BON. (a-d) Serum analysis of mice ALT, AST, ALP and ALB markers for liver function evaluations. (e) H&E staining of the liver tissues. Data are shown as mean ± SD; n=4 per group. Student's t-test was used to calculate p-values by SPSS software. *p<0.05, **p<0.01, ***p<0.001.

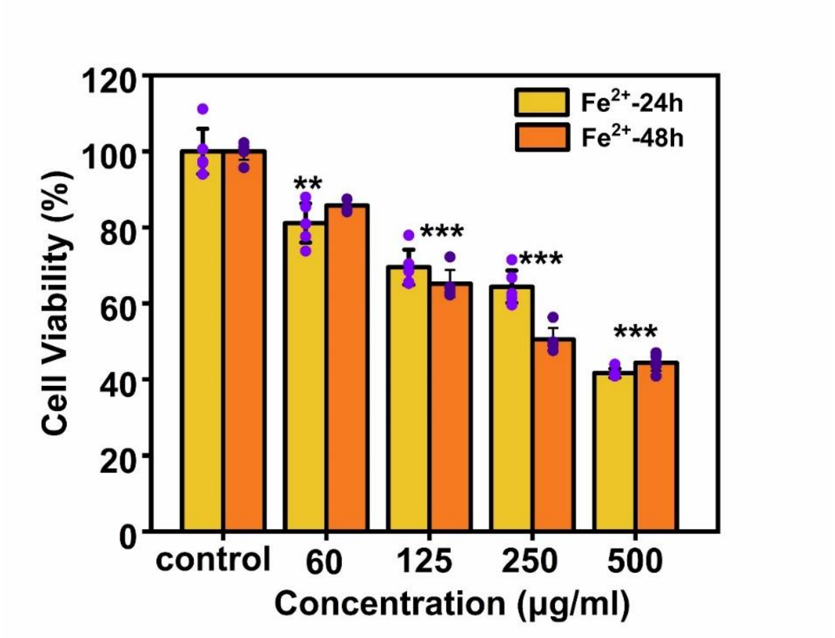

Figure S11. Cell viability of 4T1 cells co-cultured with different concentrations of Fe^{2+} for 24 and 48 hrs. Data are shown as mean ± SD; n=5 per group. Student's t-test was used to calculate p-values by SPSS software. *p<0.05, **p<0.01, ***p<0.001. Cell viability experiments for ferrous ions showed that with an increase in Fe^{2+} concentrations, the survival rate of cells decreased. However, with an increase in incubation time, there was no significant difference in cell viability between 24 and 48 hrs. In contrast, the cell viability co-incubated with BON decreased from 31% to 18% after incubation for 24 hrs compared to 48 hrs. This result indicates that, compared with the traditional Fe^{2+} catalyst, the BON nanozyme has more durable catalytic activities for more potent suppressions of cancer cells.

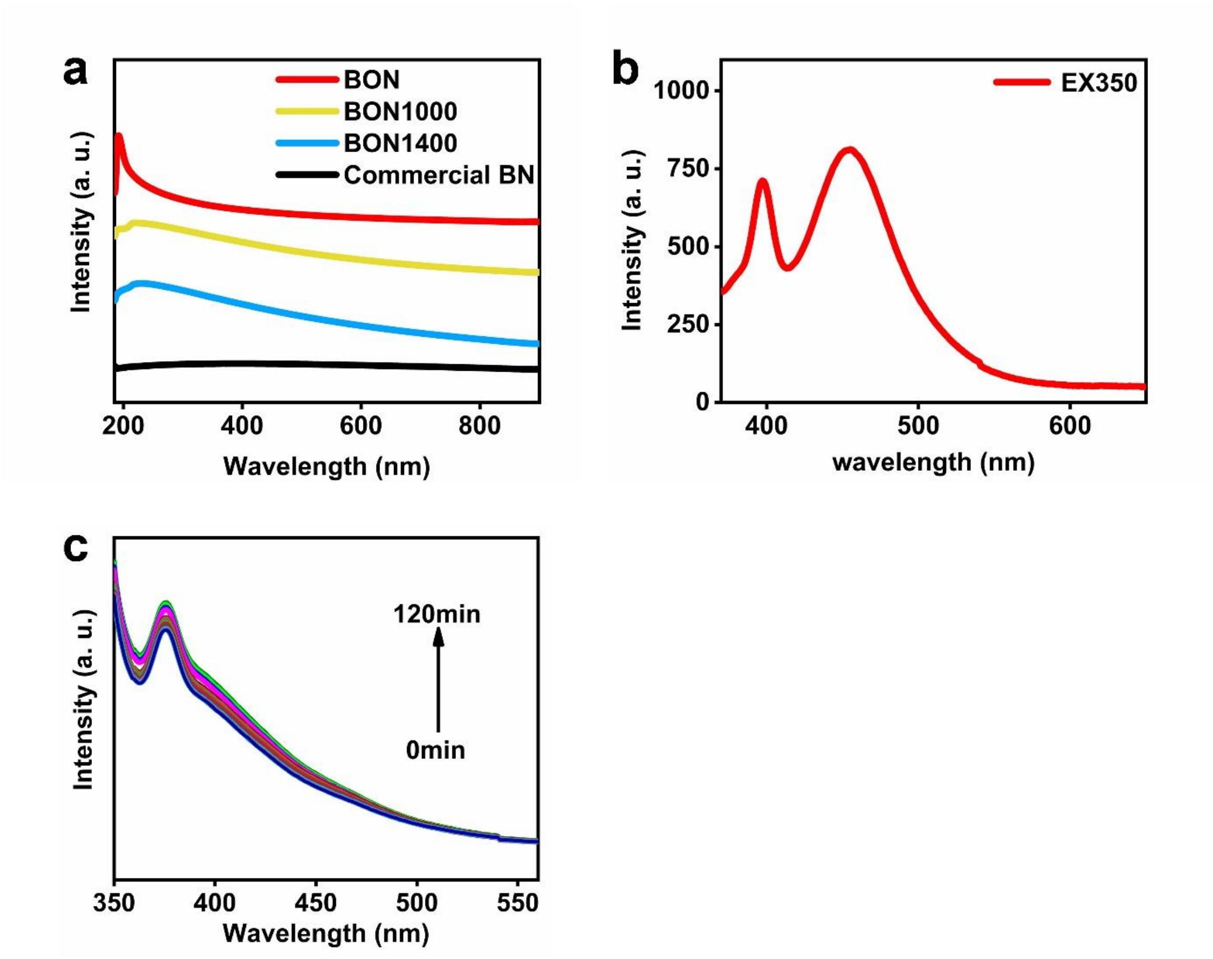

Figure S12. (a) UV spectra of BON, BON1000, BON1400, and commercial *h*-BN. (b) Fluorescence spectra of BON excited by a 350 nm light. (c) Fluorescence spectra of BON at the optimal excitation wavelength within 2 hrs. BON nanospheres are blue fluorescent under the excitation of ultraviolet light. Under continuous illuminations for 120 min, the fluorescence of BON has no significant changes, suggesting the good photostability of the BON material.

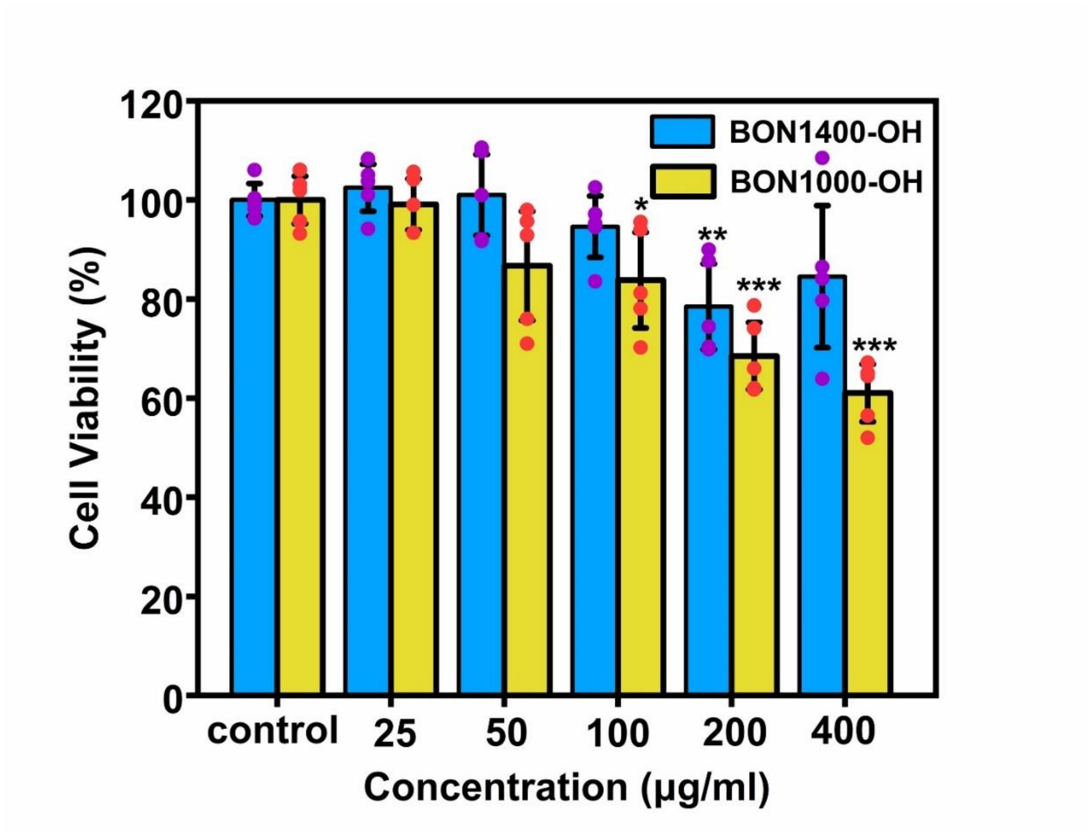

Figure S13. Cytotoxicity of hydroxylated BON1000 and BON1400 after co-culture with 4T1 cells for 24 hrs. Data are shown as mean ± SD; n=5 per group. Student's t-test was used to calculate p-values by SPSS software. *p<0.05, **p<0.01, ***p<0.001. To probe the possible catalytic centers in the BON structure, the BON1000 and BON1400 samples were treated with nitric acid for surface hydroxylation. After incubation of the hydroxylated BON1000 and BON1400 with the 4T1 cells, it was found that there were no significant differences in cell viabilities between hydroxylated samples with the regarded pristine samples, indicating that the B-OH is not the catalytic site.

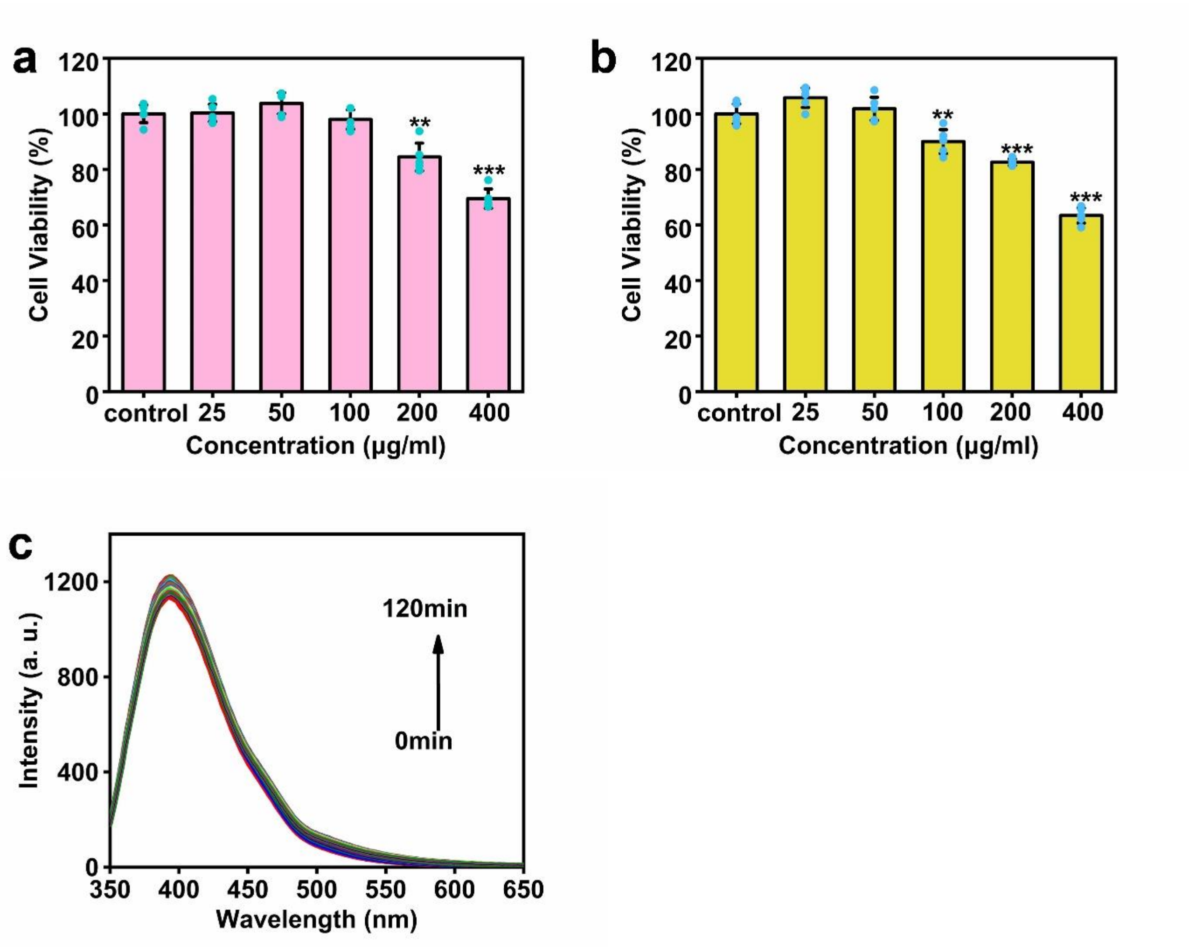

Figure S14. (a) Cytotoxicity of the eluates after 24-hrs dialysis of BON. (b) Cytotoxicity of the eluates of BON1000 after 24-hrs dialysis. (c) Fluorescence spectrum evolutions in the detection of ·OH radicals catalyzed by the BON dialysate. *Ex* = 332 nm. Data in (a,b) are shown as mean ± SD; n=5 per group. Student's t-test was used to calculate p-values by SPSS software. *p<0.05, **p<0.01, ***p<0.001.

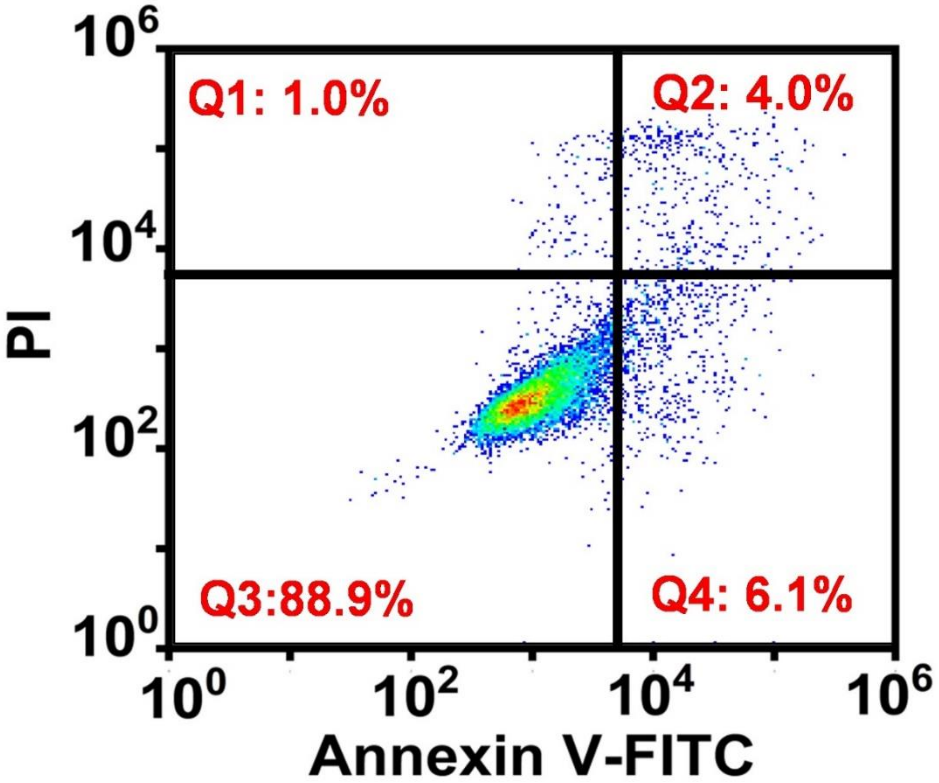

Figure S15. Flow cytometry with Annexin V-FITC/PI assay of 4T1 cells after 24 hrs co-incubation with the BON dialysate after 24-hrs dialysis of 400 μg/ml BON solution.

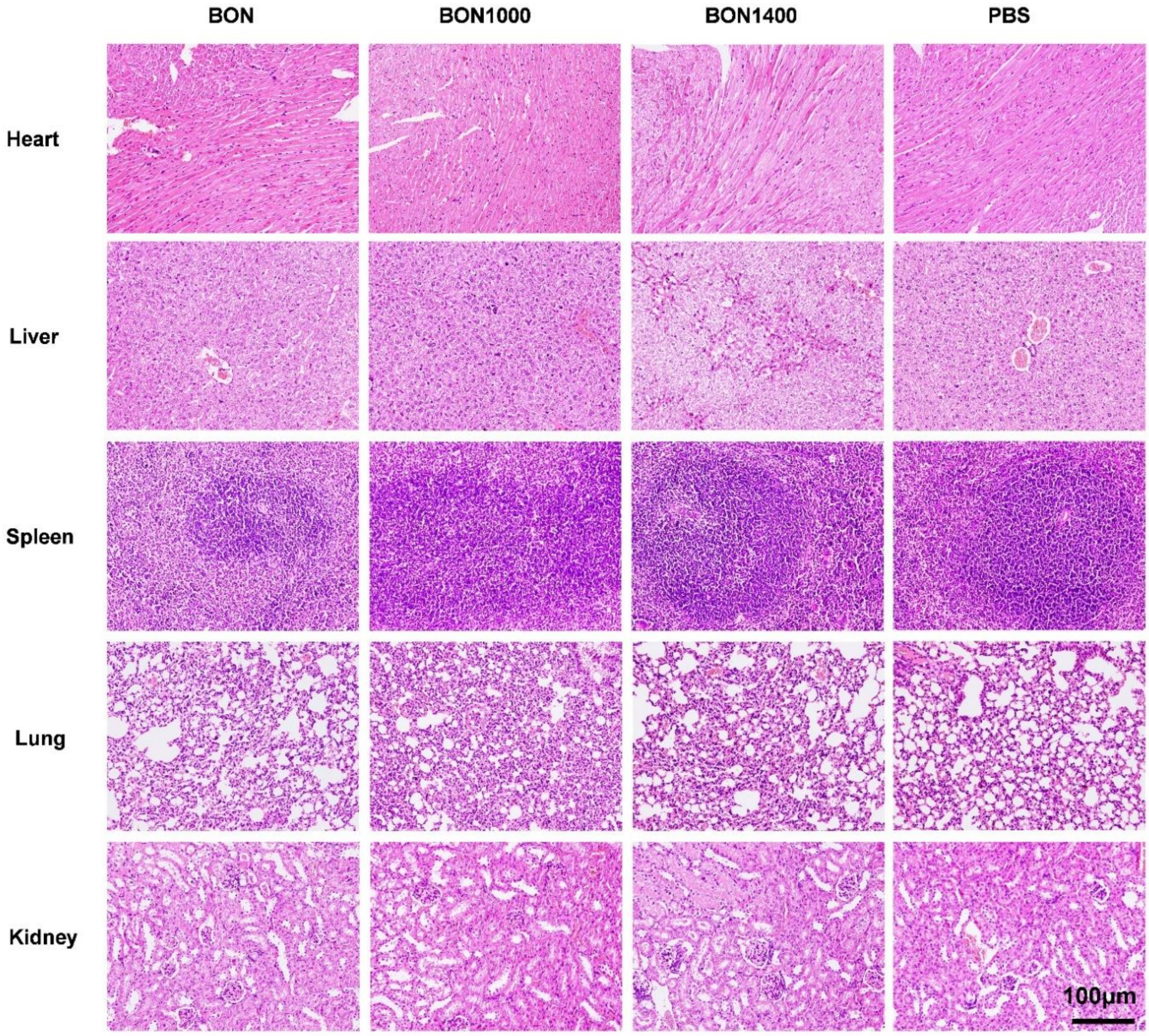

Figure S16. H&E staining of organs after different material treatments. All the images were taken at the same magnification.
